# Einthoven dissertation prizes 2013

**DOI:** 10.1007/s12471-014-0555-7

**Published:** 2014-04-10

**Authors:** E. E. van der Wall, V. A. W. M. Umans

**Affiliations:** 1 Interuniversity Cardiology Institute of the Netherlands (ICIN) - Netherlands Heart Institute, Catherijnesingel 52, P.O. Box 19258, 3501 DG Utrecht, the Netherlands; 2 Department of Cardiology, Medical Center Alkmaar, Wilhelminalaan 12, 1815 JD Alkmaar, the Netherlands

For the 25th time in a row the Interuniversity Cardiology Institute of the Netherlands (ICIN-Netherlands Heart Institute) and the Netherlands Society of Cardiology (NVVC) supported the competition for the best three cardiovascular PhD theses, published in the year 2013 [1, 2]. The dissertation prize carries the name of one of the greatest Dutchmen in the history of cardiovascular medicine, Willem Einthoven, who in 1902 for the first time recorded the human ECG, for which he received the Nobel Prize in 1924.

This time the jury received a total of 33 PhD dissertations published in 2013. The jury members were very much impressed by the high scientific quality of the PhD fellows. The ultimate selection was based on a combination of several parameters: the curriculum vitae of the candidate, the scientific originality of the PhD thesis and its relevance for the cardiovascular field. In addition, several objective bibliometric parameters were used: 1) the number of articles in first-rate journals both in PubMed and the Web of Science (WOS), 2) the number of citations in WOS, 3) the Hirsch Index, and 4) the contribution as a first author (or shared first author). Based on a combination of these results, the jury finally selected three nominees: Dr. Peter Damman (Academic Medical Centre, Amsterdam), Dr. Loek van Heerebeek (VU University Medical Centre, Amsterdam), and Dr. Rutger-Jan Nuis (Erasmus Medical Center, Rotterdam).

The members of the jury were: J.W. Deckers (Director CVOI), A. Mosterd (Chairman WCN), M.J. Schalij (Chairman Concilium NVVC), V.A. Umans (President NVVC), and E.E. van der Wall (Director ICIN).

The three candidates presented their PhD theses at the annual Spring Congress of the NVVC at the RAI Congress Centre, in Amsterdam, on Friday 4 April 2014. The ultimate winners of the first (Loek van Heerebeek), second (Peter Damman) and third prize (Rutger-Jan Nuis) were chosen by the audience. Summaries of the three nominated PhD theses are given below.

Address of correspondence:

Ernst E. van der Wall, MD

Interuniversity Cardiology Institute of the Netherlands (ICIN) - Netherlands Heart Institute, Catherijnesingel 52, PO Box 19258, 3501 DG Utrecht, the Netherlands

e-mail: ernst.van.der.wall@icin.nl

Victor A.W.M. Umans, MD

Department of Cardiology, Medical Center Alkmaar, Wilhelminalaan 12, 1815 JD Alkmaar, the Netherlands

e-mail: v.a.w.m.umans@mca.nl

## Diastolic heart failure: putting the puzzle together!

Background: Heart failure with preserved ejection fraction (HFpEF), or diastolic HF (DHF) is widely prevalent and has a poor prognosis. Its pathophysiology is incompletely understood and therapeutic strategies remain uncertain. Characteristic for DHF is high diastolic left ventricular (LV) stiffness, which is attributed to myocardial fibrosis and elevated cardiomyocyte stiffness (passive force; F_passive_). Cardiomyocyte F_passive_ is mainly determined by the elastic sarcomeric protein titin through shifts in expression of its stiff (N2B) and compliant (N2BA) isoforms and isoform phosphorylation. Whether DHF and HF with reduced EF (HFrEF) or systolic HF (SHF) represent distinct HF phenotypes is still debated.

Rationale: To support the clinical distinction between SHF and DHF, the present thesis comparatively analysed structure and function of LV myocardium procured from both DHF and SHF patients as well as from patients referred for aortic valve replacement because of severe aortic stenosis (AS). In addition, the importance of metabolic risk factors, such as diabetes mellitus type II (DMII), for diastolic LV dysfunction was investigated.

Methods: All patients were free from obstructive coronary disease. We used a translational ‘bed-to-bench’ approach, relating clinical parameters to structural, functional and biochemical characteristics of the myocardium and cardiomyocytes through procurement of LV endomyocardial biopsies, performed transvascularly in DHF (*n* = 36) and SHF (*n* = 43) patients and perioperatively in AS patients (*n* = 67). Biopsies showed no evidence of infiltrative or inflammatory myocardial disease. Biopsies were analysed for structural and biochemical characteristics using histomorphometry, electron microscopy, immunohistochemistry and gel electrophoresis, respectively. Single cardiomyocytes were isolated from the biopsies, permeabilised and stretched to 2.2 μm sarcomere length for force measurements, including cardiomyocyte F_passive_.

Results: DHF patients had a higher prevalence of obesity and more frequently had hypertension (compared with SHF) and DMII (compared with AS). Concentric LV remodelling and hypertrophy were observed in both DHF and AS patients with similarly increased cardiomyocyte diameter, in contrast to eccentric remodelling in SHF. Cardiomyocyte filamentary density was higher in DHF than in SHF, whereas interstitial myocardial fibrosis was similar in DHF and SHF, but increased in AS. Diabetes increased diastolic LV stiffness in all groups through distinct mechanisms with increased fibrosis and advanced glycation endproduct deposition contributing relatively more in SHF and increased cardiomyocyte F_passive_ contributing more in DHF. Cardiomyocyte F_passive_ was prominently higher in DHF than in SHF and AS, whereas both protein kinases A and G (PKA, PKG) acutely lowered cardiomyocyte F_passive_, through phosphorylation of titin N2B. The larger fall in F_passive_ after PKA and PKG administration in DHF implies a relative phosphorylation deficit of titin N2B in DHF. Indeed, myocardial cyclic guanosine monophosphate (cGMP) expression and PKG activity were profoundly lower in DHF than in SHF and AS, which related to higher myocardial nitrosative/oxidative stress in DHF. In DHF, downregulation of nitric oxide-cGMP-PKG signalling with subsequent hypophosphorylation of titin and increased cardiomyocyte stiffness could represent a novel mechanism contributing to elevated diastolic LV stiffness in DHF, possibly associated with the high prevalence of metabolic risk factors in DHF.

Conclusion: DHF and SHF are distinct HF phenotypes because of unequal myocardial structure and function.

*Loek van Heerebeek.*

*Department of Physiology, VUMC*

*E-mail: l.vanheerebeek@vumc.nl*

## Treatment strategies and risk stratification in acute coronary syndromes

### Summary

The first part of the thesis concerns treatment strategies for acute coronary syndromes (ACS).

For patients presenting with non-ST-elevation (NSTE)-ACS, two treatment strategies have been compared extensively in the past decade: a routine invasive strategy (routine angiography and revascularisation) and a selective invasive or conservative strategy (angiography if optimal medical treatment fails).

One chapter includes the long-term outcomes of the ‘Invasive versus Conservative Treatment in Unstable coronary Syndromes’ (ICTUS) trial in which these strategies were compared. At 5-year follow-up, we could not demonstrate a benefit of a routine invasive strategy in reducing death or myocardial infarction (MI) in patients with NSTE-ACS and elevated troponin T, irrespective of the patients’ baseline risk profile.

These data were pooled with two other large clinical trials in which these two strategies were compared: FRISC-II and RITA-3. In the patient-pooled data of these trials (the FRISC II-ICTUS-RITA-3: FIR collaboration), we showed that a routine invasive strategy reduces long-term cardiovascular death or MI and the largest absolute effect was seen in higher-risk patients. The discrepancy between the above results is probably explained by the intensity of revascularisation in the treatment arms. A high intensity of revascularisation was observed in the selective treatment arm in ICTUS, while a relatively low intensity was observed in the selective treatment arm in FIR.

After the publication of these results, the routine invasive strategy was endorsed the highest level of recommendation for high-risk patients in European and American clinical guidelines.

Using the Swedish national SWEDEHEART database to assess trends in the implementation of these treatment strategies, we observed an increase in the use of a routine invasive strategy in NSTE-ACS patients over a 12-year course.

An important point in the discussion on treatment strategies is the ‘early hazard’ of the routine invasive strategy, composed of procedure-related MIs. Using the FIR database and a critical appraisal of literature, we described that these procedure-related MIs probably do not have large clinical consequences, in contrast to spontaneously occurring MI.

The second part of the thesis concerns risk stratification in acute coronary syndromes.

In patients with NSTE-ACS, we have shown the prognostic value of qualitative assessment of the baseline electrocardiogram. Furthermore, the prognostic value of the novel biomarker growth-differentiation factor 15 (GDF-15) for long-term outcomes is described.

In patients with ST-elevation MI (STEMI), we describe that the combination of different biomarkers (glucose, estimated glomerular filtration rate and N-terminal pro-brain natiuretic peptide) improves prognostication. Furthermore, the use of a simple risk score based on these biomarkers identifies a high-risk subgroup for mortality. The simple risk score was validated in an independent database from Groningen, the Netherlands. In order to further explore the association between the risk score and higher mortality risk, we assessed the association between this score and several cardiovascular mechanistic markers of outcomes. The multimarker risk score was associated with angiographic, electrocardiographic and cardiac magnetic resonance mechanistic markers of outcomes, and our data show the ability to identify patients at high-risk of sub-optimal reperfusion and cardiac magnetic resonance outcomes who might benefit most from adjuvant treatments in STEMI.

*P. Damman*

*Department of Cardiology, Academic Medical Center - University of Amsterdam, Amsterdam, the Netherlands, e-mail:* *P.Damman@amc.uva.nl*

## Transcatheter aortic valve implantation: Current results, insights and future challenges

Aortic stenosis is a common valvular heart problem of which its prevalence increases with age. Advanced age and associated comorbidities often render these patients less amenable to surgical aortic valve replacement (AVR). In the quest for development of less invasive therapies for high-risk patients with cardiac valve disease, catheter-based aortic valve implantation (TAVI) via a peripheral blood vessel was introduced in 2004 as a novel treatment modality. Despite its advantages, TAVI remains an invasive procedure used in high-risk patients which is invariably associated with substantial risks as demonstrated in this thesis.

**Fig. 1:**
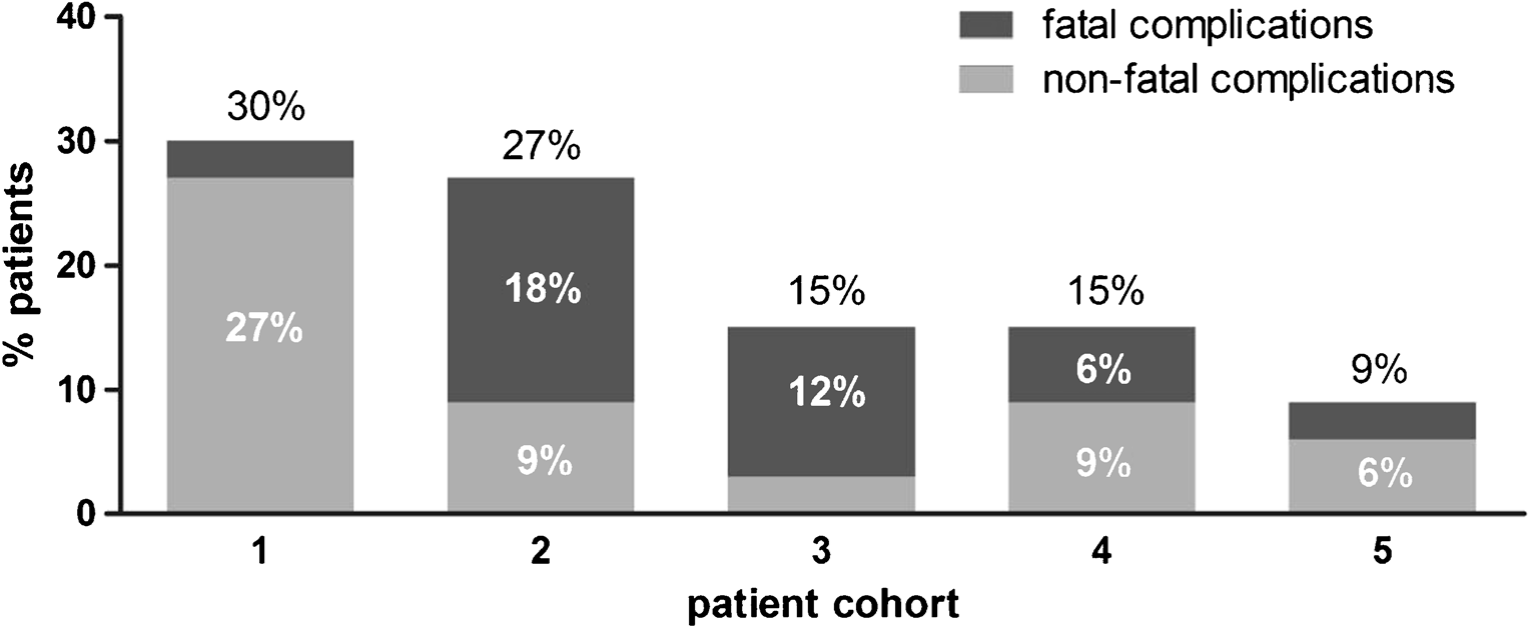
Frequencies of combined safety endpoind* at 30 days with a distinction in fatal and non-fatal complications according to 5 subsequent patient cohorts of 33 patients each. *Composite all-cause mortality, major stroke, life-threatening bleeding, acute kidney injury - stage 3, peri-procedural myocardial infraction, repeat procedure for valve-related dysfunction (surgical of interventional)

Our studies show that TAVI is associated with ≥1 (non)cardiovascular and/or permanent pacemaker implantation in 59 % of the patients while TAVI is truly uncomplicated in 41 %. Nevertheless, complication rates declined as a result of experience, and improvements in technique and technology (Fig. 1). Conduction abnormalities constitute a frequent problem during TAVI and often lead to permanent pacemaker implantation. Using continuous 12-lead ECG analyses we found that >80 % of the patients developed a new conduction defect during or after TAVI of which more than half occurred specifically during balloon pre-dilation (i.e. before actual valve implantation). The data suggest that new conduction abnormalities—and potentially also pacemaker implantation rates—can substantially be reduced by maintaining a balloon/annulus ratio of ≤1.0.

Stroke/transient ischaemic attack was seen in 9 % of the patients and half of events occurred >24 h after the procedure at a median of 5 days post-TAVI. Strokes occurring ≤24 h after TAVI were associated with balloon post-dilation of the implanted valve and valve dislodgment while strokes occurring >24 h after TAVI were associated with new-onset atrial fibrillation. Therefore, this complication may be reduced by improved postoperative care (adequate response in case of new-onset atrial fibrillation) and, eventually the use of filters placed in the cerebral arteries that capture embolic debris that is liberated during the procedure.

Acute kidney injury (AKI) occurred in 21 % of the patients following TAVI of whom 2 % needed temporary haemodialysis. Blood transfusion therapy was the strongest predictor of AKI while both transfusion and AKI predicted early and late mortality. Interestingly, patients with baseline anaemia (57 % of the cohort) had less blood loss but received more blood transfusions in comparison with patients without anaemia before TAVI, suggesting either an unconscious or better control of haemostasis during TAVI in patients with baseline anaemia and/or a lower threshold for administering transfusion during TAVI in such patients. Therefore, TAVI outcomes may be improved by a more restrictive use of transfusions, which should be directed by more strict guidelines for blood transfusion therapy in addition to optimisation of haemoglobin levels before TAVI.

The ongoing cycle of iterations in design, increase in clinical experience and ongoing clinical research assessing the role of TAVI relative to AVR but also observational cohort research in large series of patients who are more close to most patients seen in clinical practice will lead to ongoing improvements in outcome. Critical assessment in accordance with clinical and scientific principles will boost the future of TAVI to the benefit of care of patients with valvular heart disease. The technique will not be restricted to the aortic valve.

*R-J. Nuis*

*Department of Cardiology, Erasmus MC, Rotterdam, the Netherlands, e-mail:* *r.nuis@erasmusmc.nl*

**References**

1. van der Wall EE, Schalij MJ, Umans V, van Gilst WH. Einthoven dissertation prizes 2011. Neth Heart J. 2012;20:240–4. doi: 10.1007/s12471-012-0280-z.

2. van der Wall EE, van Gilst WH, Schalij MJ, Umans V. Einthoven dissertation prizes 2012. Neth Heart J. 2013;21:256–61. doi: 10.1007/s12471-013-0404-0.

